# Patients with NSCLC may display a low ratio of p.T790M *vs.* activating EGFR mutations in plasma at disease progression: implications for personalised treatment

**DOI:** 10.18632/oncotarget.20947

**Published:** 2017-09-15

**Authors:** Marzia Del Re, Paola Bordi, Iacopo Petrini, Eleonora Rofi, Francesca Mazzoni, Lorenzo Belluomini, Enrico Vasile, Giuliana Restante, Francesco Di Costanzo, Alfredo Falcone, Antonio Frassoldati, Ron H.N. van Schaik, Christi M.J. Steendam, Antonio Chella, Marcello Tiseo, Riccardo Morganti, Romano Danesi

**Affiliations:** ^1^ Clinical Pharmacology and Pharmacogenetics Unit, Department of Clinical and Experimental Medicine, University of Pisa, Pisa, Italy; ^2^ Medical Oncology Unit, University Hospital of Parma, Parma, Italy; ^3^ Medical Oncology Unit, Department of Translational Research and New Technologies in Medicine and Surgery, University Hospital of Pisa, Pisa, Italy; ^4^ Medical Oncology Unit, University Hospital Careggi, Firenze, Italy; ^5^ Medical Oncology Unit, Civil Hospital Arcispedale S. Anna, Ferrara, Italy; ^6^ Department of Clinical Chemistry, Erasmus University Medical Center, Rotterdam, The Netherlands; ^7^ Department of Pulmonology, Erasmus University Medical Center, Rotterdam, and Amphia Hospital, Breda, The Netherlands; ^8^ Lung Diseases Unit, Azienda Ospedaliero-Universitaria, Pisa, Italy; ^9^ Section of Statistics, Department of Clinical and Experimental Medicine, University of Pisa, Pisa, Italy

**Keywords:** circulating tumor DNA, EGFR, personalized medicine, predictive biomarkers

## Abstract

**Introduction:**

NSCLC harboring activating mutations of EGFR is highly sensitive to first-line EGFR-tyrosine kinase inhibitors (TKIs), but drug resistance depending on the EGFR mutation p.T790M will occur in about 50-60% of patients. Detailed information on the amount of p.T790M plasmatic level associated with resistance to EGFR-TKIs and guidance to treatment with p.T790M-effective TKI depending on these levels, is lacking.

**Methods:**

This study enrolled p.T790M-positive patients (n=49) affected by EGFR-mutated NSCLC at progression to first-line EGFR-TKIs and, in selected cases (n=5), after second-line treatment with osimertinib. Cell-free circulating tumor DNA (cftDNA) was extracted from plasma and the quantitative analysis of EGFR ex19del, p.L858R and p.T790M was performed by digital droplet PCR.

**Results:**

The mean amount of mutated alleles at progression to first-line EGFR-TKIs was 108,492 copies/ml for ex19del, 97,336 copies/ml for p.L858R, but only 8,754 copies/ml for p.T790M. There was no significant correlation between progression-free survival and the ratio of p.T790M over EGFR activating mutations. The analysis of cftDNA in 5 patients treated with osimertinib revealed a marked decrease of all EGFR mutant alleles.

**Conclusions:**

The amount of p.T790M in plasma can be much lower than activating EGFR mutations. Despite this finding, osimertinib is effective in p.T790M-positive patients. These results indicate that clones driving resistance to EGFR-TKIs represent a minority among cells bearing activating EGFR-mutations. In addition, the identification of a threshold level of p.T790M is not a strict requirement for the selection of patients to be treated with osimertinib, since treatment showed a decrease in all EGFR mutated cells.

## INTRODUCTION

Clonal heterogeneity of non-small cell lung cancer (NSCLC) has been well documented [1, 2] and contributes to the acquired resistance to targeted treatments [3-5]. Heterogeneity is a major challenge for the development of selective pharmacologic treatments. However, it remains unclear to which degree treatment outcome is affected by clonal evolution. There are multiple evidences that cells driving resistance in NSCLC are selected during treatment with EGFR-tyrosine kinase inhibitors (TKIs) [6]. Several mechanisms have been described, including the gatekeeper EGFR mutation p.T790M, MET amplification, and HER2 mutations [6-9]. The missense p.T790M is the substitution of a threonine (T) with a methionine (M) in codon 790; it is found in approximately 50-60% of NSCLCs after progression to EGFR-TKIs [10] and is the principal biomarker of resistance to EGFR-TKIs [7, 11, 12]. The p.T790M mutation is located within the gatekeeper residue of EGFR; it increases the affinity of the receptor to ATP [7] and strongly reduces the pharmacologic activity of first generation EGFR-TKIs. This view is supported by the observation that introduction of vectors carrying p.T790M into cells confers gefitinib resistance [13]. Approximately 0.32% to 78.95% of patients with NSCLC harboring EGFR activating mutations display p.T790M before administration of EGFR-TKI, although this percentage is variable according to test sensitivity [14]. As expected, subjects with a high p.T790M mutation burden had poorer clinical outcomes to EGFR-TKIs than patients with a low one [15]. It is therefore hypothesized that the selective pressure of EGFR-TKIs may select and favor the growth of p.T790M sub-clones, leading to acquired resistance.

Although data on pretreatment p.T790M [16] and at progression to first-line EGFR-TKI [11, 17-20] are available, the information on the relationship between p.T790M levels and activating EGFR mutation in patients progressing to first-generation EGFR-TKI are lacking. The availability of a minimally invasive approach based on the analysis of cell-free circulating tumor DNA in plasma (cftDNA) may represent a suitable approach to interrogate tumor heterogeneity by capturing DNA released from multiple tumor sites [4, 8] and may dynamically monitor the molecular evolution of drug resistance. Detection of p.T790M in combination with clinical signs of disease progression would represent a critical information to guide the administration of p.T790M-active drugs, including osimertinib that represents the new standard of care on this group of NSCLC patients [21, 22].

The aim of the present study was to document the relationship between plasma levels of activating EGFR mutations and p.T790M at the time of progression to first-line EGFR TKIs in patients with NSCLC and provide initial information on the dynamics of cftDNA changes upon treatment with osimertinib.

The results show that the amount of p.T790M can be substantially lower than EGFR activating mutations in drug resistance setting. Despite this, patients may respond well to osimertinib, thus a threshold value (i.e., copies/ml) of p.T790M is not required for successful treatment with p.T790M-selective drug.

## RESULTS

**Figure 1:**
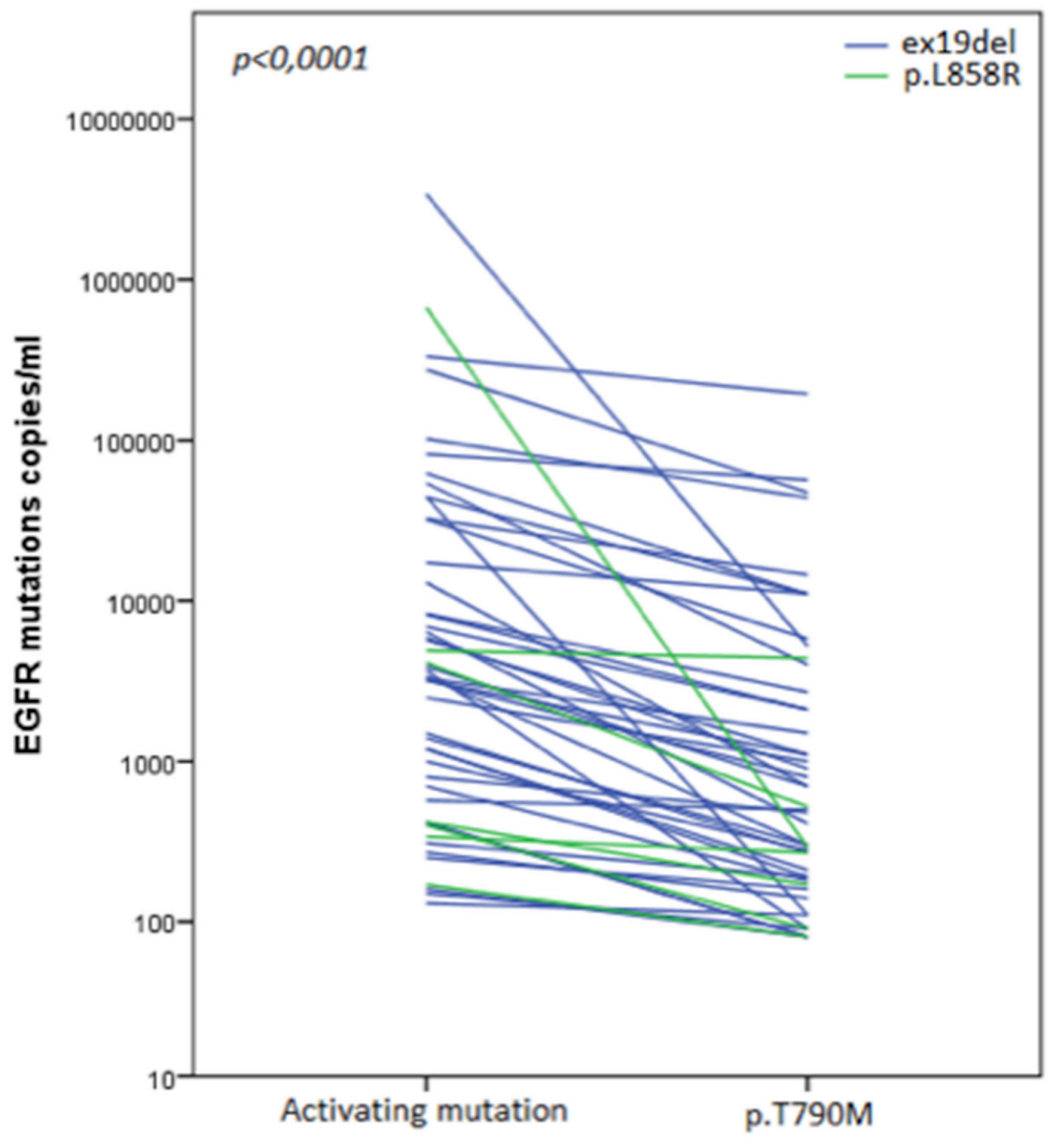
Plasma levels of EGFR activating mutations (ex19del and p. L858R) and p.T790M at the moment of disease progression to first/second-generation EGFR-TKI The analysis showed the very low ratio of p.T790M vs. activating mutations in patients at the time of progression to EGFR-TKI.

A total of 49 patients were enrolled in this study; their characteristics are summarized in Table 1. The median PFS of the first-line EGFR-TKI treatment was 21 months (range 6-57 months). The activating mutations detected in cftDNA at the time of first-line EGFR-TKI progression were ex19del (42 subjects, 85.7%) and p.L858R (7 patients, 14.3%). The number of copies/mL of plasma of EGFR activating and p.T790M mutations are reported in Table 2; the median values of ex19del, p.L858R and p.T790M were 3,550 (range 130-3,390,000), 420 (range 170-671,000) and 500 copies/mL (range 80-194,000) respectively. The median value of the ratio p.T790M/EGFR activating mutations was 0.26 (range, 0.0004-0.9) showing, overall, a low to very low ratio of p.T790M *vs.* activating mutations in patients at the time of progression to first/second-line EGFR-TKI (p<0,0001, Figure 1).

**Table 1:** Characteristics of patients

Characteristic	Statistics
**Nr. Patients**	49
**Age (years)**	63 (42 - 81)
**Sex**	
Male	16 (32.6%)
Female	33 (67.3%)
**Smoking history**	
Former	20 (40.8%)
Never	29 (59.2%)
**Stage**	
Stage IV	33 (67.3%)
Stage IIIb	16 (32.5%)
**Prior TKIs**	
Gefitinib	31 (63.3%)
Erlotinib	14 (28.6%)
Afatinib	4 (8.2%)

**Table 2:** Plasma levels of EGFR activating mutations and p.T790M

Patient	Mutation	Activating mutation (copies/ml)	p.T790M (copies/ml)	p.T790M/activating mutation ratio	Lines of treatment	PFS to EGFR-TKIs (months)
**1**	**ex19del**	333,000	194,000	0.58	1	16
**2**	**ex19del**	3,400	300	0.09	1	15
**3**	**ex19del**	1,200	190	0.16	1	35
**4**	**ex19del**	5,900	900	0.15	2	25
**5**	**ex19del**	160	80	0.5	1	12
**6**	**ex19del**	3,200	800	0.25	1	13
**7**	**ex19del**	570	500	0.87	2	27
**8**	**ex19del**	310	190	0.61	2	20
**9**	**ex19del**	1,500	270	0.18	1	12
**10**	**ex19del**	13,000	700	0.05	1	17
**11**	**ex19del**	420	80	0.19	2	23
**12**	**ex19del**	6,400	410	0.06	1	19
**13**	**ex19del**	410	80	0.19	2	11
**14**	**ex19del**	2,500	1,000	0.4	1	32
**15**	**ex19del**	700	180	0.26	3	57
**16**	**ex19del**	3,900	700	0.18	1	11
**17**	**ex19del**	32,600	14,500	0.44	2	19
**18**	**ex19del**	150	90	0.6	3	41
**19**	**ex19del**	6,900	2,100	0.3	1	23
**20**	**ex19del**	275,000	47,200	0.17	1	28
**21**	**ex19del**	54,000	4,000	0.07	1	7
**22**	**ex19del**	82,000	56,400	0.69	2	22
**23**	**ex19del**	32,100	5,800	0.18	1	13
**24**	**ex19del**	5,700	1,100	0.19	2	48
**25**	**ex19del**	130	110	0.85	1	17
**26**	**ex19del**	1,000	280	0.28	2	32
**27**	**ex19del**	44,200	11,000	0.25	1	30
**28**	**ex19del**	250	160	0.64	1	19
**29**	**ex19del**	270	140	0.52	2	28
**30**	**ex19del**	800	480	0.6	2	36
**31**	**ex19del**	3,390,000	5,200	0.0015	1	11
**32**	**ex19del**	3,700	90	0.02	1	13
**33**	**ex19del**	8,200	2,100	0.26	1	36
**34**	**ex19del**	44,400	110	0.002	1	10
**35**	**ex19del**	1,200	210	0.18	1	21
**36**	**ex19del**	3,200	1,100	0.34	1	18
**37**	**ex19del**	3,300	1,500	0.45	1	15
**38**	**ex19del**	1,400	300	0.21	1	6
**39**	**ex19del**	8,300	2,700	0.32	1	13
**40**	**ex19del**	62,000	11,000	0.17	2	10
**41**	**ex19del**	17,300	11,100	0.64	1	14
**42**	**ex19del**	102,000	43,900	0.43	1	12
**43**	**L858R**	4,120	620	0.15	2	17
**44**	**L858R**	420	170	0.4	1	10
**45**	**L858R**	400	90	0.23	2	10
**46**	**L858R**	4,900	4,400	0.9	1	11
**47**	**L858R**	170	80	0.48	1	18
**48**	**L858R**	671,000	290	0.0004	1	13
**49**	**L858R**	340	270	0.8	2	20
**Median (range)**		3,400 (130-3,390,000)	500 (80-194,000)	0.26 (0.0004-0.9)		17 (6-57)

**Figure 2:**
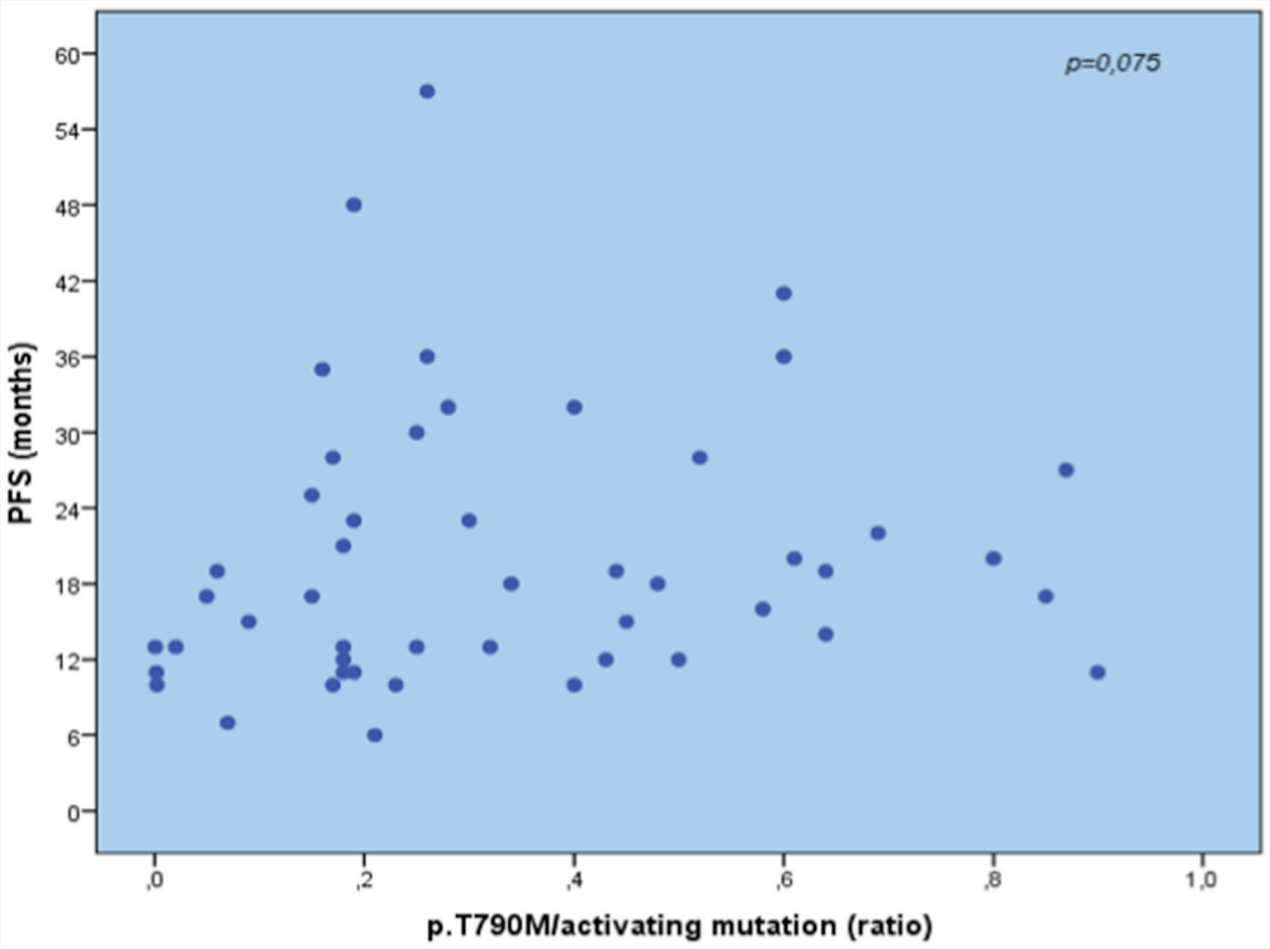
Absence of correlation between p. T790M/activating mutation ratio and PFS to first/second-generation EGFR-TKI, highlighting that a high ratio is not required to obtain resistance to treatment, but also very low amounts drive the resistance

The statistical analysis of PFS *vs.* the ratio of p.T790M/EGFR activating mutations showed no significant difference (p = 0.075; Spearman’s rank correlation coefficient = 0.256) suggesting that previous EGFR-TKI does not influence the ratio p.T790M/EGFR and that a high ratio is not required to obtain resistance to treatment (Figure 2). No differences in mutation amounts were seen between fast and slow progressing patients comparing months of PFS.

**Figure 3:**
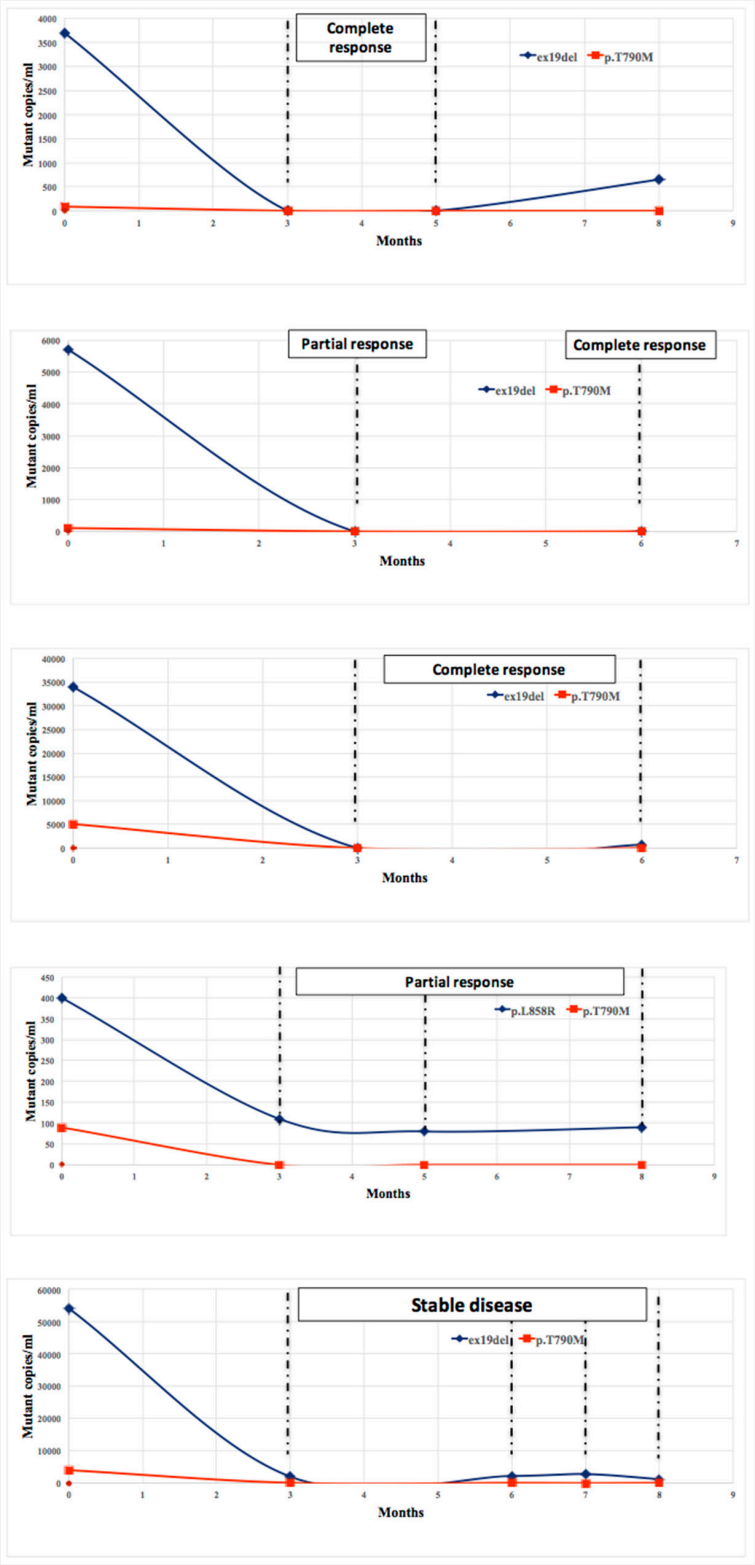
Decreasing of mutated EGFR alleles (ex19del, p. L858R, p.T790M) in plasma during treatment with osimertinib The amount of EGFR mutant clones in plasma rapidly decreased in parallel and was maintained during treatment, accordingly to the tumor response.

The amount of ex19del, p.L858R and p.T790M EGFR mutant copies in plasma was monitored during treatment with osimertinib in 5 patients (2 with complete response to osimertinib, 2 with partial response and 1 with stable disease at first-tumor evaluation after 12 weeks) in order to gather information about the dynamics of EGFR mutational pattern as a function of therapy and time. The amount of EGFR mutant clones in plasma decreased in parallel (ex19del and p.T790M or p.L858R and p.T790M), although the activity of osimertinib is higher on p.T790M than ex19del and p.L858R and it would be, therefore, expected a steeper decline of p.T790M than ex19del and p.L858R. In general, the reduction of both ex19del and p.T790M was more marked than in the single patient bearing both p.T790M and p.L858R, which persisted in plasma (Figure 3). The decrease of mutated alleles in plasma was maintained during response to treatment.

## DISCUSSION

The EGFR gatekeeper mutation p.T790M confers resistance to first- and second- generation EGFR-TKIs and prolongs the biologic condition of tumor addiction to EGFR transduction pathway in these tumors. Therefore, the development of new drugs with improved targeting capability of EGFR activating mutations and with extended spectrum to p.T790M is an active area of research [23, 24]. Osimertinib is the first drug approved for the treatment of p.T790M-positive NSCLC after failure of first/second generation EGFR TKIs [21, 22].

Detection of mutant alleles can be performed in tissue as well as plasma, and ddPCR is a sensitive technology suitable for cftDNA detection and analysis [25]. Indeed, our previous work demonstrated that ddPCR detection of p.T790M in plasma reached a meaningful sensitivity of 81.8% and a specificity of 85.7%; in addition, the overall concordance between plasma and tissue analysis was good and corresponded to 83.3% [8].

Although the detection of p.T790M in EGFR-mutant NSCLC patients can be performed on both plasma and tumor tissue, the evolution of diagnostic approaches towards minimally-invasive procedures such as cftDNA is desirable for a number of reasons, including a) the invasive character of a tissue biopsy, b) unreachable tumor sites or insufficient tissue obtained after biopsy [26] and c) limitation of tissue biopsy in capturing tumor heterogeneity due to the small amount of tissue collected and number of tumor sites sampled. Indeed, ESMO clinical practice guidelines for diagnosis, treatment and follow-up of patients with NSCLC reports that if the p.T790M in peripheral blood is observed, treatment with third-generation EGFR TKIs is justified, while it recommends rebiopsy if cftDNA is negative for p.T790M [27]. Timely analysis of biomarkers to guide treatment decision is crucial and any delay in obtaining molecular testing results can postpone treatment decisions and reduce effectiveness of therapy for patients with advanced NSCLC [28].

p.T790M can be detected in plasma at a median time of 2.2 months prior to disease progression and is a predictive factor of resistance and disease outcome [29]. The role of p.T790M may change, depending on disease stage/phase. In particular, patients positive for p.T790M before treatment with EGFR-TKI show significantly inferior PFS (8.9 vs. 12.1 months) and overall survival (19.3 vs. 31.9 months) compared with those without p.T790M [30]. On contrary, at the time of disease progression after first- and second- generation EGFR-TKI, the presence of EGFR p.T790M is a favourable prognostic marker independently from the treatment with osimertinib [11]. Patients with p.T790M had a significantly longer post-progression survival, while subjects without p.T790M more often progressed in a previously uninvolved organ system and exhibited a poorer performance status at time of progression [11].

p.T790M shows a complex biological behaviour, since p.T790M status in patients may change both temporally and spatially among tumor sites at least in part due to the selective pressure from EGFR-TKI [31]. Interestingly, p.T790M status of NSCLC varies after EGFR-TKI discontinuation and may change from positive to negative, thus justifying a potential re-challenge with EGFR-TKI [31]. It is not surprising that after further EGFR-TKI administration, p.T790M status may change from negative to positive again [31]. Previous work provided initial evidence of the different amounts of p.T790M vs. activating mutations in plasma samples in 9 patients [32]. In addition, a recent report suggested a possible clinical importance for detection of p.T790M at low levels in plasma samples [18]. Longitudinal cftDNA analysis revealed an increase in plasma EGFR-activating mutation, and p.T790M announced rociletinib resistance in some patients, whereas in others the activating mutation increased but p.T790M remained suppressed [20]. These findings demonstrate the role of tumor heterogeneity when drugs targeting a single resistance mechanism are given to patients [20]. Based on the results of the present study, it would be interesting to evaluate the effectiveness of combination regimens that also target p.T790–wild-type clones. The present work shows that patients progressing to first/second-generation EGFR-TKIs displayed a low amount of p.T790M compared to the EGFR activating mutations.

On the basis of the cftDNA data of the present work it can be speculated that either EGFR amplification could be a frequent finding in these patients, or that p.T790M is present in a minority of cells of the tumor cell population after TKI failure or both. Therefore, the intriguing questions are: 1) how frequent is EGFR amplification in these patients, 2) how p.T790M can drive tumor progression being so low within the tumor; 3) is there another mechanism of resistance in addition to p.T790M, and 4) is p.T790M able to synergize with activating EGFR mutation.

Our data indicate that, even in presence of low amount of p.T790M, tumors respond to the third-generation TKI osimertinib, which is mainly active against p.T790M. The drug shows higher activity against the EGFR double mutants ex19del/p.T790M and p.L858R/p.T790M with respect to the EGFR bearing only the activating mutation as demonstrated by *in vitro* experiments. In particular, mean IC_50_ values are 17 and 4 nM for the ex19del and the p.L858R, respectively and 13 and 5 nM for the double mutants ex19del+p.T790M and p.L858R+p.T790M, respectively [6]. These data are confirmed also in patients of the present study because by monitoring the amount of residual EGFR mutations during osimertinib treatment, it is clear that cell clones carrying the double mutant ex19del/p.T790M or p.L858R/p.T790M and clones with the ex19del alone drastically decrease in their amount, while cell clones carrying the p.L858R alone remain detectable because of the low potency of osimertinib against this mutation.

In patients monitored during osimertinib treatment, p.T790M reduction was marked and similar to the decline of plasma levels of EGFR activating mutations. The differences in concentration between them and the p.T790M may confirm that the tumor is heterogeneous, and it is composed by 1) wild-type clones, 2) cells carrying both the EGFR activating and p.T790M mutations, 3) clones with only the original EGFR activating mutation. A distinct population of cells with the p.T790M only is unlikely to be present.

In conclusion, the present work demonstrates the feasibility of detecting very low amounts of p.T790M in plasma by a sensitive analytical approach and that this mutation is associated with tumor progression – and response to osimertinib – even though it may be a minority with respect to ex19del and p.L858R activating mutations. Further studies are warranted to gain additional knowledge on the interaction between EGFR mutations in TKI-resistant NSCLC, and to determine the clinical consequences to be connected to cftDNA outcomes in plasma. However, nowadays, is often required a treatment adaptation based on pharmacogenetic data [33, 34], and these results provide strong evidence supporting the usefulness of cftDNA as a good predictive biomarker.

## MATERIALS AND METHODS

The present study included 49 NSCLC patients carrying the p.T790M mutation in cftDNA. These patients were consecutively enrolled between June and October 2016 from a population of subjects with the following characteristics: 1) stage IIIb/IV disease carrying EGFR activating mutations (exon 19 deletions [ex19del] and/or exon 21 p.L858R) in tumor tissue at the time of initial diagnosis; 2) treatment with first- or second-generation EGFR-TKIs (gefitinib/erlotinib/afatinib), as per approved indication and 3) clinical and imaging evidence of disease progression, as per standard practice.

In order to detect EGFR ex19del, p.L858R and p.T790M mutations, one blood sample was taken in each patient at the time of disease progression; in 5 subjects (enrolled in ASTRIS Trial [35]), 2 to 4 additional blood drawings were obtained to monitor EGFR ex19del, p.L858R and p.T790M mutations during osimertinib administration.

Blood was sampled as per routine biochemistry testing, collected in EDTA tubes and centrifuged at 4°C for 10 min at 3000 rpm within two hours after drawing. Plasma samples (2 ml) were taken from material to be discarded and stored at −80°C until analysis. Circulating tumor DNA was extracted using the QIAmp Circulating Nucleic Acid Kit (Qiagen®, Valencia, CA, USA) following the manufacturer’s protocol. The ex19del, p.L858R and p.T790M alleles were examined using a digital droplet PCR (QX100™ Droplet Digital™ PCR System, BioRad®, Hercules, CA, USA) as previously described [8]. Droplets with a fluorescence intensity threshold higher than 3,000 were considered positive for the presence of mutations and results were given as copies of allele/ml.

This study was compliant with local ethical practices; in particular, patients received treatments as per approved drug label and molecular tests were performed on residual material from standard diagnostic procedures.

### Statistical analysis

Progression-free survival (PFS) was calculated from the first day of first-line EGFR-TKI treatment to radiological evidence of disease progression according to RECIST criteria; deaths for other causes than disease progression were considered events [36]. PFS of patients receiving osimertinib was not considered for statistical analysis.

Before inferential statistics, an exploration phase was performed using box plots and scatter plots. We assessed three main quantitative factors: 1) concentration of the activating mutations (ex19del, p.L858R), 2) levels of p.T790M, and 3) their ratio (resistant/activating). In order to verify if the quantitative data were normally distributed we used the Kolmogorov–Smirnov and Shapiro–Wilks tests. A comparison between the three factors and PFS (all patients relapsed to first-line EGFR-TKI) was performed by a nonparametric correlation analysis (Spearman’s rank correlation coefficient), whereas Kruskal–Wallis and Mann–Whitney (two-tailed) tests were used to perform comparisons among the factor associated to the different treatment lines. Finally, the Wilcoxon test (two-tailed) has been performed to compare the number of copies/ml of plasmatic EGFR activating and p.T790M mutations. A p<0.05 was considered as statistically significant; all statistical analyses were performed using the SPSS version 24 software.
